# P2Y12 Inhibitor Monotherapy: Considerations for Acute and Long-Term Secondary Prevention Post-PCI

**DOI:** 10.31083/j.rcm2310348

**Published:** 2022-10-17

**Authors:** Antonio Greco, Maria Sara Mauro, Davide Capodanno, Dominick J. Angiolillo

**Affiliations:** ^1^Division of Cardiology, Azienda Ospedaliero-Universitaria Policlinico “G. Rodolico – San Marco”, 95125 Catania, Italy; ^2^Division of Cardiology, University of Florida College of Medicine, Jacksonville, FL 32209, USA

**Keywords:** acute coronary syndrome, antiplatelet therapy, antithrombotic therapy, chronic coronary syndrome, percutaneous coronary intervention, P2Y_12_ receptor, pharmacotherapy, secondary prevention

## Abstract

Following percutaneous coronary intervention (PCI), an initial course of dual antiplatelet therapy (DAPT) with aspirin and a ${\mathrm{P2Y}}_{12}$ inhibitor (${\mathrm{P2Y}}_{12}$-i) is recommended to minimize the risk of thrombotic complications. After the initial period of DAPT, antiplatelet monotherapy, usually consisting of aspirin, is administered for long-term secondary prevention. However, over the last few years there has been accruing evidence on ${\mathrm{P2Y}}_{12}$-i monotherapy, both in the acute (i.e., post-PCI; after a brief period of DAPT, transitioning to monotherapy before six or 12 months in patients with chronic or acute coronary syndrome, respectively) and chronic (i.e., long-term secondary prevention; after completion of six or 12 months of DAPT, in patients with chronic or acute coronary syndrome, respectively) settings. In aggregate, most studies of short DAPT with transition to ${\mathrm{P2Y}}_{12}$-i monotherapy showed a reduced risk of bleeding complications, without any significant increase in ischemic events as compared to standard DAPT. On the other hand, the evidence on long-term ${\mathrm{P2Y}}_{12}$-i monotherapy is scarce, but results from a randomized trial showed that clopidogrel monotherapy outperformed aspirin monotherapy in terms of net benefit, ischemic events and bleeding. Antiplatelet therapy is also recommended for patients undergoing PCI and with an established indication for long-term oral anticoagulation (OAC). In this scenario, a brief period of triple therapy (i.e., aspirin, ${\mathrm{P2Y}}_{12}$-i and OAC) is followed by a course of dual antithrombotic therapy (usually with ${\mathrm{P2Y}}_{12}$-i and OAC) and ultimately by lifelong OAC alone. European and American guidelines have been recently updated to provide new recommendations on antithrombotic therapy, including the endorsement of ${\mathrm{P2Y}}_{12}$-i monotherapy in different settings. However, some areas of uncertainty still remain and further randomized investigations are ongoing to fulfil current gaps in knowledge. In this review, we assess the current knowledge and evidence on ${\mathrm{P2Y}}_{12}$-i monotherapy for the early and long-term secondary prevention in patients undergoing PCI, and explore upcoming research and future directions in the field.

## 1. Introduction

Initial observations of platelets in the human blood date back to the 19th century, when Max Schultze and Giulio Bizzozero [1, 2] afterward identified and described the role of what appeared as unknown blood spherules, both *in vitro *and *in vivo*. Platelets were then found to play a central role in thrombosis and hemostasis, adhering to one another and to some threads later recognized as strands of fibrin [3]. Platelets became a therapeutical target in the 1960s, when the effects of aspirin on bleeding time were correlated to impairment in platelet response [4]. Approximately 30 years later it became clear that platelets can be also activated by different stimuli, including the ${\mathrm{P2Y}}_{12}$ receptor pathway [5].

${\mathrm{P2Y}}_{12}$ is a 7-membrane-spanning receptor coupled to an inhibitory G protein that binds adenosine 5’ diphosphate (ADP) and is essential for a normal platelet response [6]. Indeed, a rare inherited ${\mathrm{P2Y}}_{12}$ receptor deficiency is associated with impaired platelet aggregation and a propensity to bleed [7]. The ${\mathrm{P2Y}}_{12}$ receptor also participates to additional functions, such as stabilization of platelet aggregates mediated by thrombin or thromboxane $A_{2}$ (${\mathrm{TXA}}_{2}$), reduction of cytokines production, mitigation of airway inflammation in allergic asthma, and antitumoral response [6]. Different classes of ${\mathrm{P2Y}}_{12}$ receptor inhibitors (${\mathrm{P2Y}}_{12}$-i) are currently available, in both oral and intravenous formulations, and feature different pharmacologic profiles but with the common clinical indication consisting in the treatment and secondary prevention of atherosclerotic disease manifestations [6]. Indeed, percutaneous coronary intervention (PCI) has represented a key area for the development and clinical use of oral ${\mathrm{P2Y}}_{12}$-i. A detailed description of the pharmacologic profiles of ${\mathrm{P2Y}}_{12}$-i goes beyond the scope of this manuscript. In brief, clopidogrel, prasugrel and ticagrelor are the three most commonly utilized oral ${\mathrm{P2Y}}_{12}$-i, which will be referred to for the purpose of this review.

Following PCI, irrespective of whether in the context of a patients presenting with a chronic coronary syndrome (CCS) or acute coronary syndrome (ACS), an initial course of dual antiplatelet therapy (DAPT) with aspirin and a ${\mathrm{P2Y}}_{12}$-i (usually six months for CCS and 12 months for ACS patients) is recommended to minimize the risk of thrombotic complications [8, 9, 10]. However, DAPT conveys an unavoidable risk of bleeding. Importantly, bleeding complications have an adverse impact on short- and long-term prognosis, underscoring the need for bleeding reduction strategies [11, 12]. Shortening the duration of DAPT represents an important bleeding reduction strategy [13, 14]. Although shortening DAPT duration has traditionally consisted in discontinuing the ${\mathrm{P2Y}}_{12}$-i while maintaining aspirin monotherapy, most recently there has been accruing evidence supporting discontinuation of aspirin with transition to ${\mathrm{P2Y}}_{12}$-i monotherapy [15, 16, 17, 18]. In addition, ${\mathrm{P2Y}}_{12}$-i monotherapy is also emerging as a treatment strategy for long-term secondary prevention, a field where aspirin monotherapy has for decades represented the standard of care [17, 18, 19, 20].

This article reviews the current evidence on ${\mathrm{P2Y}}_{12}$-i monotherapy for early and long-term secondary prevention of cardiovascular events in patients undergoing PCI.

## 2. Mechanism of Action

**Fig. 1.**
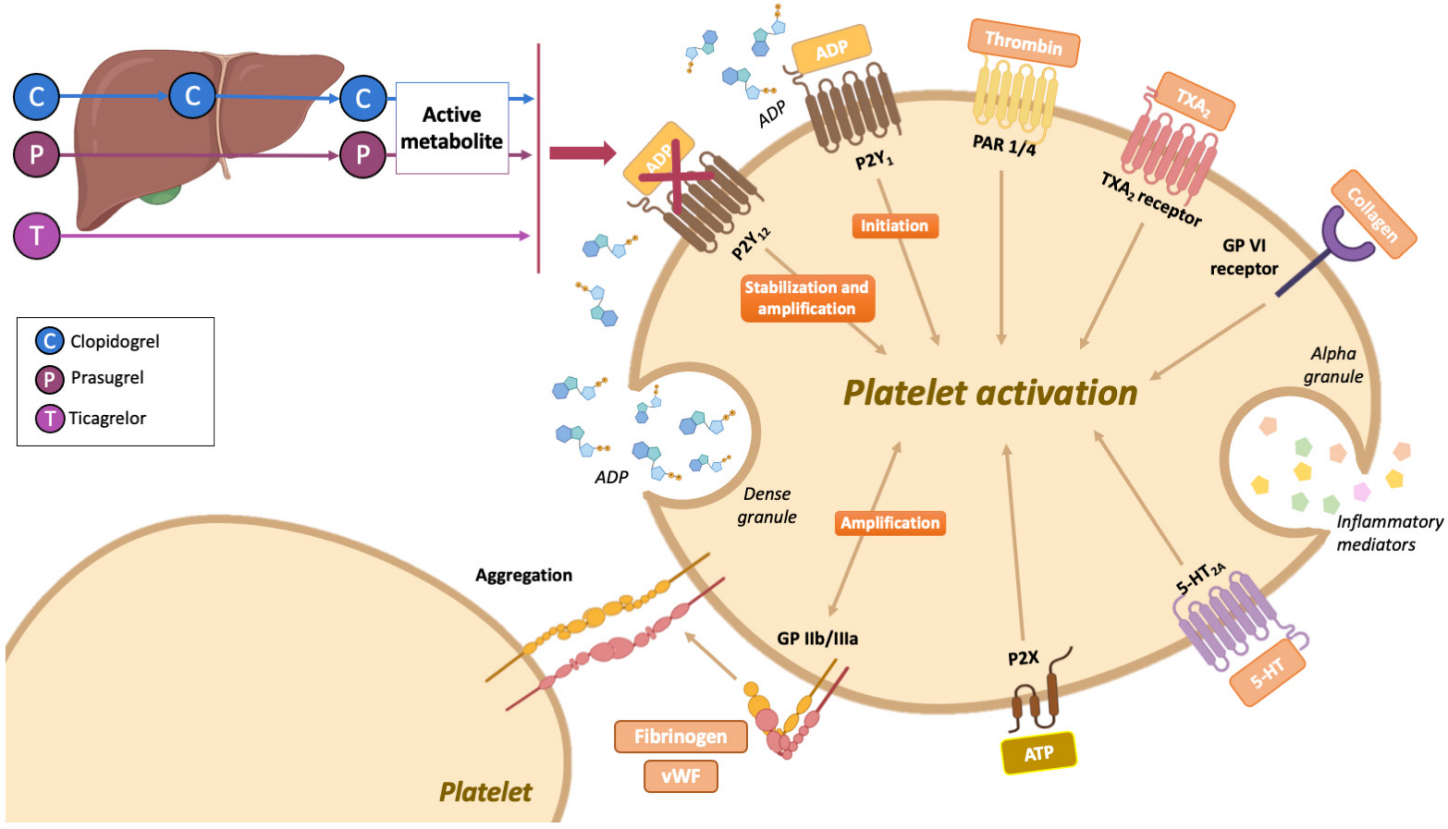
**Mechanism of action of oral ${\mathrm{P2Y}}_{12}$-inhibitors**. The mechanism of action of oral ${\mathrm{P2Y}}_{12}$-inhibitors consists of the blockage of the platelet ${\mathrm{P2Y}}_{12}$ receptor, which is a 7-membrane-spanning receptor from the P2 family. The ${\mathrm{P2Y}}_{12}$ receptor is normally activated by the adenosine 5’ diphosphate (ADP) released from dense granules following platelet activation and is coupled to an inhibitory G protein that inhibits adenylyl cyclase, translating into the induction of platelet aggregation. The main role of the ${\mathrm{P2Y}}_{12}$ receptor is to amplify platelet activation (also supported by signaling via the glycoprotein IIb/IIIa receptor), which however requires the ${\mathrm{P2Y}}_{1}$ receptor for the initiation phase. Different drugs can inhibit the ${\mathrm{P2Y}}_{12}$ receptor: clopidogrel and prasugrel require a two- and one-step, respectively, hepatic biotransformation to generate active metabolites that irreversibly inhibit the ${\mathrm{P2Y}}_{12}$ receptor. On the other hand, ticagrelor is directly active (i.e., does not require hepatic metabolism to exert its pharmacological activity, although 30% of its effects is attributed to a hepatic-derived metabolite) and reversibly binds to the ${\mathrm{P2Y}}_{12}$ receptor. Abbreviations: 5-HT, 5-hydroxytryptamine; ${\mathrm{5HT}}_{\text{2A}}$, 5-hydroxytryptamine receptor 2A; ADP, adenosine 5’ diphosphate; ATP, adenosine triphosphate; C, clopidogrel; GPIIb/IIIa, glycoprotein IIb/IIIa; GP VI, platelet glycoprotein VI; P, prasugrel; PAR, proteinase-activated receptor; T, ticagrelor; ${\mathrm{TXA}}_{2}$, thromboxane $A_{2}$; vWF, von Willebrand factor.

Aspirin and ${\mathrm{P2Y}}_{12}$-i block different pathways of platelet activation (Fig. 1).

Three nucleotide receptors (jointly known as P2 receptors), namely ${\mathrm{P2X}}_{1}$, ${\mathrm{P2Y}}_{1}$ and ${\mathrm{P2Y}}_{12}$, can be triggered by the ADP released following platelet activation from dense granules, where it is stored at high concentrations; platelet activation is initiated by the ${\mathrm{P2Y}}_{1}$ receptor and requires the ${\mathrm{P2Y}}_{12}$ for amplification and sustainment of the process; in case of blockade of the ${\mathrm{P2Y}}_{12}$ receptor, ${\mathrm{P2Y}}_{1}$ mediates a small and rapidly reversible platelet aggregation [21]. Oral ${\mathrm{P2Y}}_{12}$-i include reversible (i.e., ticagrelor) or irreversible (i.e., clopidogrel and prasugrel) agents that block the binding of ADP to the ${\mathrm{P2Y}}_{12}$ receptor (e.g., clopidogrel and prasugrel) or ADP-induced signal transduction (e.g., ticagrelor). Clopidogrel and prasugrel are thienopyridines that are pro-drugs, hence requiring two- and one-step hepatic conversion into the active metabolite, respectively, to generate an active metabolite; conversely, ticagrelor is a cyclopentyltriazolopyrimidine that is directly active, although 30% of its effects is attributed to a hepatic-derived metabolite [21]. Table 1 summarizes clinically approved and investigational ${\mathrm{P2Y}}_{12}$-i.

**Table 1.** **Pharmacological profiles of ${\mathrm{P2Y}}_{12}$ inhibitors**.

	Ticlopidine	Clopidogrel	Prasugrel	Ticagrelor	Cangrelor	Vicagrel	Selatogrel
Class	Thienopyridine	Thienopyridine	Thienopyridine	Cyclopentyltriazolopyrimidine	Adenosine triphosphate analogue	Thienopyridine	2-phenyl-pyrimidine-4-carboxamide analogue
Binding	Irreversible	Irreversible	Irreversible	Reversible	Reversible	Irreversible	Reversible
Type of binding	Noncompetitive	Competitive	Competitive	Noncompetitive	Competitive	Competitive	Competitive
Metabolic conversion	Yes	Yes	Yes	No	No	Yes	No
Route of administration	Oral	Oral	Oral	Oral	Intravenous	Oral	Subcutaneous
Dose	250 mg twice daily MD	600 mg LD, 75 mg daily MD	60 mg LD, 10 mg daily MD	180 mg LD, 90 mg twice daily MD	30 µg/kg bolus, 4 µg/kg/min infusion (two to four hours)	20 mg LD, 5 mg daily MD	16 mg
Onset of action	Two hours	Two to six hours	0.5 to four hours	0.5 to two hours	Two minutes	Four hours	15 to 30 minutes
Offset of action	Seven to 10 days	Seven to 10 days	Seven to 10 days	Three to five days	One to 1.5 hours	Five to 10 days	Eight hours
Half-life	Eight to 13 hours depending on age	AM 30 minutes	AM seven hours	AM nine to 12 hours	Three to five minutes	AM 45 minutes	Four to 7 hours
Approved for clinical use	Yes	Yes	Yes	Yes	Yes	No	No

Abbreviations: AM, active metabolite; LD, loading dose; MD, maintenance dose.

Conversely, the fundamental mechanism responsible for the antithrombotic effects of aspirin is the irreversible inhibition of cyclooxygenase-1 (COX-1), which suppresses the platelet production of ${\mathrm{TXA}}_{2}$ [22]. Synergistic inhibitory effects of aspirin and ${\mathrm{P2Y}}_{12}$-i on platelet function were initially demonstrated in studies with clopidogrel [23]. However, blocking the ${\mathrm{P2Y}}_{12}$ receptor can also hamper platelet activation and aggregation mediated by other platelet activation pathways, including ${\mathrm{TXA}}_{2}$ [24, 25]. An *in-vitro* study in low-shear conditions showed that aspirin provided only a small additional inhibitory effect in presence of a potent ${\mathrm{P2Y}}_{12}$-i blockage with the active metabolite of prasugrel [26]. Conversely, studies conducted in high-shear conditions (more similar to the *in vivo* arterial blood flow) suggested a residual role of aspirin in inhibiting collagen-induced platelet activation, even when associated with a potent ${\mathrm{P2Y}}_{12}$-i [27].

### Pharmacodynamic Studies

Pharmacodynamic studies have suggested that aspirin discontinuation is followed by increased platelet reactivity by the COX-1 pathway, while pathways depending on other agonists (e.g., ADP, ${\mathrm{TXA}}_{2}$ and thrombin receptor-activating peptide 6 [TRAP-6]) remain adequately silenced with ${\mathrm{P2Y}}_{12}$-i monotherapy, providing a mechanistic rationale to reduce bleeding while still ensuring adequate ischemic protection [28, 29, 30].

The TEMPLATE trial used a panel of platelet function tests after randomly allocating 110 ACS patients undergoing PCI to receive either ticagrelor monotherapy or DAPT with aspirin and ticagrelor for four weeks, with both strategies followed by aspirin monotherapy for additional four weeks [28]. Platelet aggregation in response to TRAP-6 (primary outcome), ${\mathrm{TXA}}_{2}$ agonism and ADP was similar between the study groups; unsurprisingly, the response to arachidonic acid was reduced only in the DAPT group due to the effects of aspirin. In the ticagrelor monotherapy group, platelet aggregation induced by a collagen-related peptide (specific agonist of the platelet glycoprotein VI receptor) was higher, suggesting an incomplete suppression of collagen-mediated platelet activation [28].

In the TWILIGHT platelet sub-study (n = 51), ticagrelor monotherapy and DAPT were compared in terms of thrombus size (primary endpoint) and platelet reactivity following different stimuli. Blood thrombogenicity (i.e., thrombus size in the *ex-vivo* Badimon perfusion chamber) was similar between the two groups as well as platelet reactivity in response to ADP and thrombin. By contrast, platelet reactivity after arachidonic acid or collagen was higher among patients receiving ticagrelor monotherapy, highlighting the unequivocal role of aspirin in the inhibition of the COX-1 pathway [29].

The GLOBAL LEADERS platelet sub-study, excluding patients on DAPT with aspirin and clopidogrel, explored the restoration of platelet reactivity after withdrawal of aspirin at one month or ticagrelor at 12 months [30]. Cessation of either component of DAPT led to a substantial increase in platelet reactivity, with differential effects depending on the specific investigated activation pathway. After aspirin withdrawal, there was a marked recovery of platelet aggregation induced by arachidonic acid or collagen; by contrast, cessation of ticagrelor was followed by a prompt recovery of platelet aggregation in response to ADP or collagen [30].

Given that most pharmacodynamic studies conducted thus far have used assays that are specific to appraise the effects of pathways inhibited by a given antiplatelet agent, more studies evaluating the diverse effects of the different antiplatelet regimens (e.g., aspirin monotherapy, ${\mathrm{P2Y}}_{12}$-i monotherapy, or DAPT) using assays able to assess the effects on global thrombogenicity (similar to the Badimon chamber) are warranted.

## 3. Evidence on Monotherapy with a ${\mathrm{P2Y}}_{12}$ Inhibitor

**Fig. 2.**
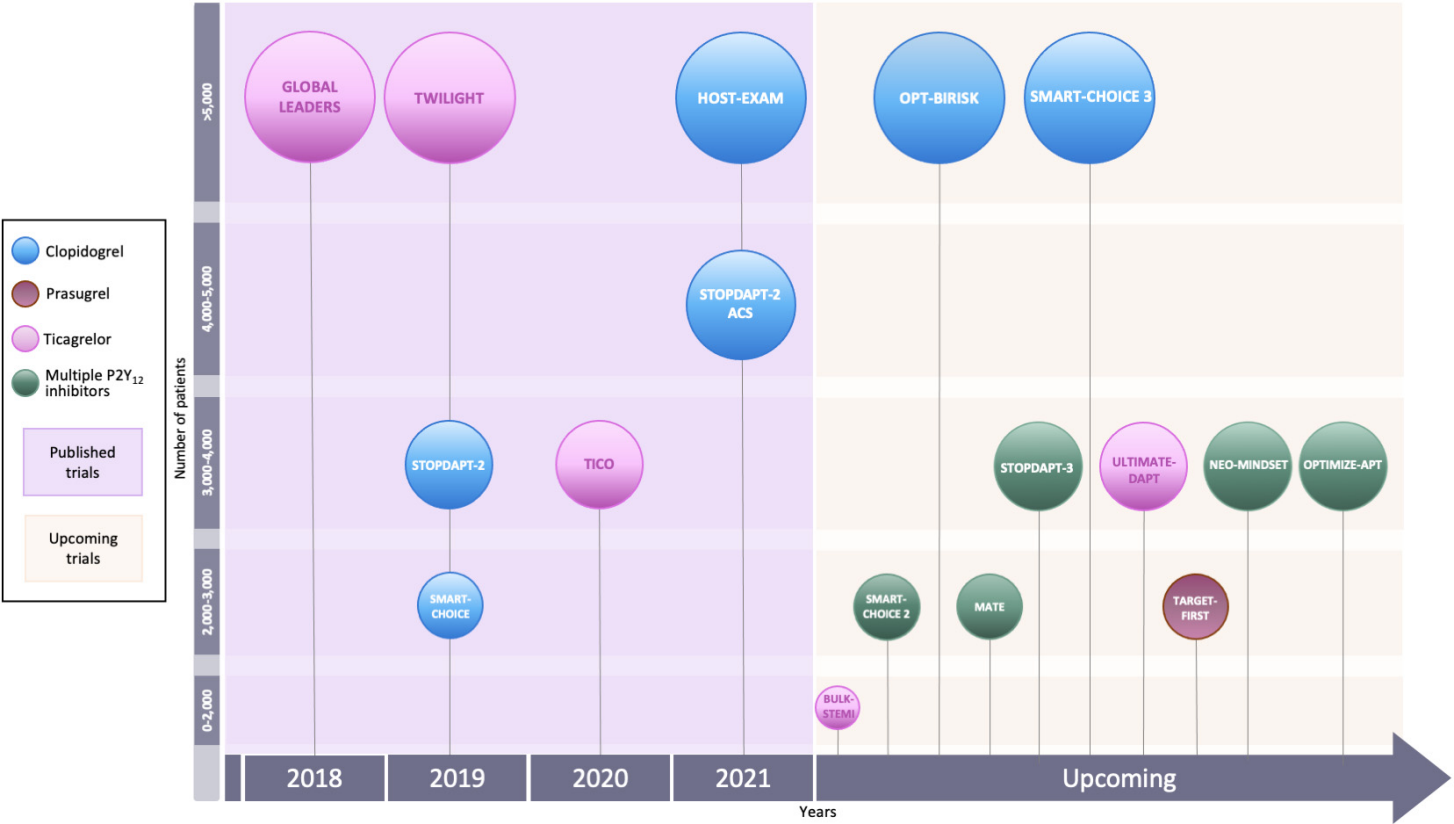
**Randomized clinical trials of ${\mathrm{P2Y}}_{12}$-inhibitor monotherapy after PCI**. Randomized clinical trials of ${\mathrm{P2Y}}_{12}$-inhibitor monotherapy after percutaneous coronary intervention are shown in according to their time of publication. Horizontal position of trials in the graphs reflects the year of publication; in particular, seven already published trials are presented in the lavender box (on the left), while nine ongoing trials are illustrated in the beige box (on the right). The diameters of the spheres are proportionate with respect to the study sample size (i.e., very small for study of less than 1000 patients; small for studies of 1000 to 2000 patients; medium for studies of 2000 to 3000 patients; large for studies of 4000 to 5000 patients; and very large for studies greater than 5000 patients). In addition, a glance on study sample size can be also appraised by their vertical position in the graph. The color of the spheres refers to the ${\mathrm{P2Y}}_{12}$-inhibitor predominant in the investigational arm of each study (i.e., blue for clopidogrel, brown for prasugrel, purple for ticagrelor, green if similar percentages of multiple ${\mathrm{P2Y}}_{12}$-inhibitors have been used).

Several randomized clinical trials (RCTs) have investigated the role of ${\mathrm{P2Y}}_{12}$-i monotherapy after a shorter or longer course of DAPT in patients undergoing PCI (Fig. 2).

### 3.1 Early ${\mathrm{P2Y}}_{12}$ Inhibitor Monotherapy

RCTs on early ${\mathrm{P2Y}}_{12}$-i monotherapy include investigations performed with different ${\mathrm{P2Y}}_{12}$-i (i.e., clopidogrel, prasugrel, ticagrelor), at different timepoints (i.e., immediately after PCI, or at one-to-three months) and using DAPT as a control.

#### 3.1.1 ${\mathrm{P2Y}}_{12}$ Inhibitor Monotherapy Three Months after PCI

RCTs investigating short DAPT followed by aspirin monotherapy showed consistent benefits in terms of bleeding mitigation as compared to standard DAPT (i.e., six to 12 months depending on the clinical setting). Although conclusive findings about ischemic protection cannot be drawn mostly due to the enrolment of low-risk patients and some lack of statistical power, meta-analyses have warned about the potential increase in the risks of myocardial infarction (MI) or stent thrombosis following early DAPT discontinuation [31, 32]. Evidence of bleeding reduction with clopidogrel monotherapy compared with DAPT in the setting of cerebrovascular disease prompted the initiation of RCTs in PCI patients to investigate DAPT shortened to three months followed by ${\mathrm{P2Y}}_{12}$-i monotherapy (Table 2) [33, 34].

**Table 2.** **Early ${\mathrm{P2Y}}_{12}$-inhibitor monotherapy: three months after PCI**.

	SMART-CHOICE	TWILIGHT	TICO
Population	East-Asian patients undergoing PCI (n = 2993)	Patients at high risk of bleeding or ischemic events undergoing PCI (n = 7119)	East-Asian patients with ACS undergoing PCI (n = 3056)
ACS	58%	65%	100%
${\mathrm{P2Y}}_{12}$ inhibitor	Clopidogrel	Ticagrelor	Ticagrelor
Randomization timing	At the time of PCI	Three months after PCI	At the time of PCI
Investigational strategy	DAPT for three months, followed by ${\mathrm{P2Y}}_{12}$-i monotherapy for nine months	${\mathrm{P2Y}}_{12}$-i monotherapy for 12 months	DAPT for three months, followed by ${\mathrm{P2Y}}_{12}$-i monotherapy for 12 months
Control strategy	DAPT for 12 months	DAPT for 12 months	DAPT for 12 months
Follow-up	Twelve months from randomization	Twelve months from randomization (i.e., 15 months from PCI)	Twelve months from randomization
Primary outcome(s)	Death, MI or stroke (difference 0.4%; one-sided 95% CI -∞ to 1.3%; *p* = 0.007 for noninferiority)	BARC type 2, 3 or 5 bleeding (HR 0.56; 95% CI 0.45 to 0.68; *p *< 0.001)	Death, MI, stent thrombosis, stroke, target-vessel revascularization or TIMI major bleeding (difference –1.98%; 95% CI –3.50% to –0.45%; HR 0.66; 95% CI 0.48 to 0.92; *p* = 0.01)
		Death, MI or stroke (difference –0.06%; 95% CI –0.97 to 0.84; HR 0.99; 95% CI 0.78 to 1.25; *p *<0.001 for noninferiority)	
Bleeding outcome	BARC type 2-5 bleeding (HR, 0.58; 95% CI, 0.36 to 0.92; *p* = 0.02)	BARC type 2, 3 or 5 bleeding (HR 0.56; 95% CI 0.45 to 0.68; *p *< 0.001)	TIMI major bleeding (HR 0.56; 95% CI 0.34 to 0.9; *p* = 0.02)

Results are presented by reporting the effect of interventional strategy versus reference treatment. Abbreviations: ACS, acute coronary syndrome; BARC, bleeding academic research consortium; CI, confidence interval; DAPT, dual antiplatelet therapy; HR, hazard ratio; MI, myocardial infarction; PCI, percutaneous coronary intervention; ${\mathrm{P2Y}}_{12}$-i, ${\mathrm{P2Y}}_{12}$ inhibitor; TIMI, thrombolysis in myocardial infarction.

The SMART-CHOICE open-label noninferiority trial randomized 2993 East Asian patients undergoing PCI to three-month DAPT followed by clopidogrel monotherapy or 12-month DAPT [35]. ${\mathrm{P2Y}}_{12}$-i monotherapy was noninferior to DAPT in terms of major adverse cardiac and cerebrovascular events (MACCEs) at 12 months (2.9% vs. 2.5%; difference 0.4%; one-sided 95% confidence interval [CI] -∞ to 1.3%; *p* = 0.07 for noninferiority) and significantly reduced the incidence of Bleeding Academic Research Consortium (BARC) type 2-5 bleeding (2.0% vs. 3.4%; hazard ratio [HR] 0.58; 95% CI 0.36 to 0.92; *p* = 0.02). There were no between-group differences in other endpoints, including stent thrombosis (0.2% vs. 0.1%; HR 1.51; 95% CI 0.25 to 9.02; *p* = 0.65). However, this trial enrolled East Asian patients only, mostly treated with clopidogrel, did not have a placebo control, and showed a high rate of non-adherence to the investigational strategy (approximately 20%) [35, 36]. Notably, the treatment effect of ${\mathrm{P2Y}}_{12}$-i monotherapy was consistent regardless of on-treatment high platelet reactivity or procedural complexity [37, 38].

TWILIGHT was a randomized double-blind RCT exploring the effect of aspirin discontinuation after three months of DAPT in patients at high risk of bleeding or ischemic events undergoing PCI [39]. After three months, 7119 patients who had not adverse events while on DAPT were randomized to receive ticagrelor plus placebo or ticagrelor plus aspirin for additional one year. The primary endpoint was BARC bleeding type 2, 3 or 5, while ticagrelor monotherapy was also tested for noninferiority to DAPT with respect to major adverse cardiovascular events (MACEs). At 12 months after randomization, ticagrelor monotherapy significantly reduced the incidence of bleeding (4.0% vs. 7.1%; HR 0.56; 95% CI 0.45 to 0.68; *p *< 0.001) and was noninferior for MACEs as compared to DAPT (3.9% vs. 3.9%; difference –0.06%; 95% CI –0.97 to 0.84; HR 0.99; 95% CI 0.78 to 1.25; *p *< 0.001 for noninferiority), without any significant difference in stent thrombosis (0.4% vs. 0.6%; HR 0.74; 95% CI 0.37 to 1.47). Subgroup analyses showed consistent results irrespective of age, sex, region of randomization, diabetes, chronic kidney disease, prior MI, high bleeding risk, PCI complexity or stent type [40, 41, 42, 43, 44, 45, 46, 47, 48]; conversely, ticagrelor monotherapy was more beneficial in ACS patients (*p* = 0.03 for interaction) [49].

Similarly, the TICO trial investigated ticagrelor monotherapy after three-month DAPT in East Asian ACS patients [50]. Differently from the TWILIGHT trial, patients (n = 3056) were randomized at the time of PCI to receive either three-month DAPT followed by ticagrelor monotherapy or 12-month ticagrelor-based DAPT. Ticagrelor monotherapy was significantly associated with lower rates of the primary endpoint of net adverse cardiovascular events (NACEs) as compared to DAPT (3.9% vs. 5.9%; difference –1.98%; 95% CI –3.50% to –0.45%; HR 0.66; 95% CI 0.48 to 0.92; *p* = 0.01), driven by a reduction in major bleeding (1.7% vs. 3.0%; HR 0.56; 95% CI 0.34 to 0.91; *p* = 0.02), without a significant difference in the risk of MACCE (2.3% vs. 3.4%; HR 0.69; 95% CI 0.45 to 1.06; *p* = 0.09) or stent thrombosis (0.4% vs. 0.3%; HR 1.51; 95% CI 0.43 to 5.33; *p* = 0.53). Results were similar in multiple subgroup analyses and in a landmark analysis between three and 12 months [50].

Despite limitations and heterogeneity in the design and conduction of these RCTs, in aggregate they showed that shortening DAPT to three months by withdrawing aspirin is associated with a reduction in bleeding as compared to standard DAPT, with no overt signals of harm with respect to ischemic or thrombotic protection, both in CCS and ACS patients.

#### 3.1.2 ${\mathrm{P2Y}}_{12}$ Inhibitor Monotherapy One Month after PCI

DAPT can be also shortened by withdrawing aspirin after only one month as investigated by three RCTs (Table 3).

**Table 3.** **Early ${\mathrm{P2Y}}_{12}$-inhibitor monotherapy: one month after PCI**.

	GLOBAL LEADERS	STOPDAPT-2	STOPDAPT-2 ACS
Population	Patients undergoing PCI (n = 15,968)	East-Asian patients undergoing PCI (n = 3045)	East-Asian patients with ACS undergoing PCI (n = 4169)
ACS	47%	38%	100%
${\mathrm{P2Y}}_{12}$ inhibitor	Ticagrelor	Clopidogrel	Clopidogrel
Randomization timing	At the time of PCI	At the time of PCI	At the time of PCI
Investigational strategy	DAPT for one month, followed by ${\mathrm{P2Y}}_{12}$-i monotherapy for 23 months	DAPT for one month, followed by ${\mathrm{P2Y}}_{12}$-i monotherapy for 11 months	DAPT for one-to-two months, followed by ${\mathrm{P2Y}}_{12}$-i monotherapy for 11 months
Control strategy	DAPT for 12 months, followed by aspirin monotherapy for 12 months	DAPT for 12 months	DAPT for 12 months
Follow-up	Twenty-four months from randomization	Twelve months from randomization	Twelve months from randomization
Primary outcome(s)	Death or Q-wave MI (rate ratio 0.87; 95% CI 0.75 to 1.01; *p* = 0.073)	Cardiovascular death, MI, stroke, stent thrombosis, or TIMI major or minor bleeding (difference –1.34%; 95% CI –2.57 to –0.11; HR 0.64; 95% CI 0.42 to 0.98; *p *< 0.001 for noninferiority; *p* = 0.04 for superiority)	Cardiovascular death, MI, stroke, stent thrombosis, or TIMI major or minor bleeding (HR 1.14; 95% CI 0.80 to 1.62; *p* = 0.06 for noninferiority)
Bleeding outcome	BARC type 3-5 bleeding (rate ratio 0.97; 95% CI 0.78 to 1.20; *p* = 0.77)	Major bleeding (absolute difference –1.13%; 95% CI –1.84% to –0.42%; HR 0.26; 95% CI 0.11 to 0.64; *p* = 0.004 for superiority)	TIMI major or minor bleeding (absolute difference –0.63%; 95% CI –1.20% to –0.06%; HR 0.46; 95% CI 0.23 to 0.94)

Results are presented by reporting the effect of interventional strategy versus reference treatment. Abbreviations: ACS, acute coronary syndrome; BARC, Bleeding Academic Research Consortium; CI, confidence interval; DAPT, dual antiplatelet therapy; HR, hazard ratio; MI, myocardial infarction; PCI, percutaneous coronary intervention; ${\mathrm{P2Y}}_{12}$-i, ${\mathrm{P2Y}}_{12}$ inhibitor; TIMI, thrombolysis in myocardial infarction.

The GLOBAL LEADERS multicenter open-label superiority RCT randomized 15,968 all-comer PCI patients to either one-month DAPT with aspirin and ticagrelor followed by ticagrelor monotherapy for 23 months or standard 12-month DAPT with aspirin and a ${\mathrm{P2Y}}_{12}$-i (clopidogrel or ticagrelor for CCS and ACS, respectively) followed by aspirin monotherapy for 12 months [51]. This design implied the theoretical distinction of the GLOBAL LEADERS trial in three periods: (i) during the first month the trial compared two DAPT regimens; (ii) between one and 12 months ticagrelor monotherapy and DAPT; (iii) between 12 and 24 months ticagrelor and aspirin monotherapies [52]. There was no significant between-group difference in terms of the primary outcome (composite of death or Q-wave MI) at 24 months (3.81% vs. 4.37%; rate ratio [RR] 0.87; 95% CI 0.75 to 1.01; *p* = 0.073) or stent thrombosis (0.8% vs. 0.8%; HR 1.00; 95% CI 0.71 to 1.42; *p* = 0.98) and with respect to BARC 3 or 5 bleeding (2.04% vs. 2.12%; RR 0.97; 95% CI 0.78 to 1.20; *p* = 0.77). Interestingly, the treatment effect on bleeding was affected by the reference treatment (*p* = 0.007 for interaction), with advantages of ticagrelor monotherapy over ticagrelor-based DAPT in ACS (1.95% vs. 2.68%; RR 0.73; 95% CI 0.54 to 0.98; *p* = 0.037) but not over a clopidogrel-based regimen in CCS (2.13% vs. 1.62%; RR 1.32; 95% CI 0.97 to 1.81; *p* = 0.081) [53]. In the landmark analysis between 30 days and two years, ticagrelor monotherapy was not associated with any benefit or harm as compared to standard therapy with respect to the primary endpoint (3.40% vs. 3.87%; RR 0.88; 95% CI 0.74 to 1.03; *p* = 0.115) and bleeding (1.43% vs. 1.54%; RR 0.93; 95% CI 0.72 to 1.20; *p* = 0.576) [51]. In a landmark analysis between 30 days and one year restricted to ACS patients, ticagrelor monotherapy numerically decreased the incidence of the primary endpoint (1.5% vs. 2.0%; HR 0.73; 95% CI 0.51 to 1.03; *p* = 0.07) and significantly reduced bleeding (0.8% vs. 1.5%; HR 0.52; 95% CI 0.33 to 0.81; *p* = 0.004) [54]. Subgroup analyses showed advantages of the investigational strategy in patients undergoing complex PCI, multivessel PCI, stenting of proximal left anterior descending or long stenting [55, 56, 57, 58], while no interaction was found with age, diabetes, or stenting of bifurcation lesions [59, 60, 61]. The GLOBAL LEADERS trial had an open-label design and was affected by asymmetrical crossover in favor of the control, potentially diluting the treatment effect, particularly in intention-to-treat analyses [52]. To address the lack of central event adjudication of study endpoints, the GLASSY adjudication sub-study included patients from the 20 top-enrolling sites (n = 7585) for central adjudication of endpoints by an independent clinical event committee, resulting in noninferiority (but not superiority) of the investigational strategy to the control group in terms of death or Q-wave MI (7.14% vs. 8.41%; RR 0.85; 95% CI 0.72 to 0.99; *p *< 0.001 for noninferiority; *p* = 0.0465 for superiority, with a one-sided type I error of 2.5%), without any difference in bleeding (2.48% vs. 2.48; RR 1.00; 95% CI 0.75 to 1.33; *p = *0.986) [62]. These results were consistent regardless of the clinical scenario [63].

The STOPDAPT-2 noninferiority RCT investigated a one-month DAPT with aspirin and clopidogrel or prasugrel followed by clopidogrel monotherapy as compared to standard DAPT with aspirin and clopidogrel for 12 months in 3045 East Asian patients undergoing PCI [64]. One-month DAPT was noninferior and also superior to standard DAPT in terms of NACE at 12 months (2.36% vs. 3.0%; absolute difference –1.34; 95% CI –2.57% to –0.11%; HR 0.64; 95% CI 0.42 to 0.98; *p *< 0.001 for noninferiority; *p* = 0.04 for superiority); clopidogrel monotherapy also overcame standard DAPT with respect to major or minor bleeding (0.41% vs. 1.54%; HR 0.26; 95% CI 0.11 to 0.64; *p* = 0.004), while being noninferior for MACE (1.96% vs. 2.51%; absolute difference –0.55%; 95% CI –1.62% to 0.52%; HR 0.79; 95% CI 0.49 to 1.29; *p* = 0.005 for noninferiority; *p* = 0.34 for superiority); finally, there was no between-group difference in the rate of stent thrombosis (0.13% vs. 0.07%; HR 2.02; 95% CI 0.18 to 22.26; *p* = 0.57) [64]. These results were confirmed by subgroup analyses in patients undergoing complex PCI, at high bleeding risk, and in carriers of CYP2C19 loss-of-function alleles [65, 66, 67, 68]. However, this trial should be interpreted in the light of several limitations, such as the use of a net benefit endpoint, the lower-than-anticipated event rates, the low statistical power for ischemic events and the eventual selective enrolment of low-risk patients.

Similarly, the STOPDAPT-2 ACS trial enrolled 4136 Japanese patients undergoing PCI due to an ACS (partially from the STOPDAPT-2 cohort) translating the same design of the STOPDAPT-2 trial to a different population. One-month DAPT failed to prove noninferior to 12-month DAPT for NACE (3.2% vs. 2.8%; absolute difference 0.37%; 95% CI –0.68% to 1.42%; *p* = 0.06 for noninferiority), with a numerical increase in MACE (2.8% vs. 1.9%; absolute difference 0.90%; 95% CI –0.02% to 1.82%; HR 1.50; 95% CI 0.99 to 2.26), particularly MI (HR 1.91; 95% CI 1.06 to 3.44), and a reduction in bleeding (0.5% vs. 1.2%; absolute difference –0.63%; 95% CI –1.20% to –0.06%; HR 0.46; 95% CI 0.23 to 0.94); there was no difference in stent thrombosis between the two groups (0.5% vs. 0.2%; HR 2.29; 95% CI 0.70 to 7.42) [69].

In the pooled STOPDAPT-2 total cohort, clopidogrel monotherapy was noninferior (and not superior) to standard DAPT in terms of net benefit (2.84% vs. 3.04%; HR 0.94; 95% CI 0.70 to 1.27; *p* = 0.001 for noninferiority; *p* = 0.68 for superiority), with a reduction in bleeding (0.50% vs. 1.31%; HR 0.38; 95% CI 0.21 to 0.70; *p* = 0.002), without a significant increase in the risk of MACE (2.40% vs. 1.97%; HR 1.24; 95% CI 0.88 to 1.75; *p* = 0.14 for noninferiority; *p* = 0.23 for superiority) [70].

Results from these trials showed that shortening DAPT to one month could be a viable option in selected patients (e.g., high bleeding risk, particularly among CCS patients), with a note of caution and more data warranted in patients with ACS.

#### 3.1.3 ${\mathrm{P2Y}}_{12}$ Inhibitor Monotherapy Immediately after PCI

There is also preliminary evidence about very early (i.e., immediately after the procedure) aspirin withdrawal in patients undergoing PCI. The only experience in this setting is represented by the ASET pilot study, which is also the only investigation of prasugrel monotherapy so far [71]. This multicenter open-label single-arm study enrolled 201 CCS patients with low anatomical complexity (i.e., SYNTAX score <23) undergoing PCI. A loading dose of prasugrel was administered immediately after PCI and aspirin was stopped on the same day; after three months, prasugrel monotherapy was associated with very low rates of MACE (0.5%) and BARC 3 or 5 bleeding (0.5%), without stent thrombosis events. This study opened to the possibility of an immediate ${\mathrm{P2Y}}_{12}$-i monotherapy in low-risk CCS patients. However, the ASET study was not randomized and its population did not display characteristics justifying an immediate withdrawal of aspirin (i.e., high bleeding risk features) nor the use of a potent ${\mathrm{P2Y}}_{12}$-i (i.e., high thrombotic risk features, such as ACS or complex PCI), eventually hindering the benefit or drawbacks of this strategy [71].

#### 3.1.4 Pooled Evidence on Shortening DAPT after PCI

Meta-analyses of the GLOBAL LEADERS, SMART-CHOICE, STOPDAPT-2, TWILIGHT and TICO trials confirmed that shortening DAPT to one or three months by discontinuing aspirin reduced the incidence of bleeding as compared with standard DAPT, without any increase in MACE, both in CCS and ACS patients [72, 73]. In addition, an individual patient-level meta-analysis of six RCTs (also including the small DACAB trial on ticagrelor monotherapy after coronary artery bypass grafting [CABG]) confirmed that ${\mathrm{P2Y}}_{12}$-i monotherapy is associated with similar rates of MACE as compared to standard DAPT (2.95% vs. 3.27%; HR 0.93; 95% CI 0.79 to 1.09; *p* = 0.005 for noninferiority; *p* = 0.38 for superiority) and reduced BARC bleeding type 3 or 5 (0.89% vs. 1.83%; HR 0.49; 95% CI 0.39 to 0.63; *p *< 0.001), particularly when prasugrel or ticagrelor were part of the reference group (*p* 0.02 for interaction) [74].

Collectively, the results of these RCTs and meta-analyses suggest that shortening DAPT to three or one month and continuing with ${\mathrm{P2Y}}_{12}$-i monotherapy is an effective bleeding mitigation strategy that does not seem to affect thrombotic or ischemic protection. However, again, a note of caution due to potential withdrawal of protection should be raised over the early use clopidogrel monotherapy in ACS.

### 3.2 Long-term ${\mathrm{P2Y}}_{12}$ Inhibitor Monotherapy

${\mathrm{P2Y}}_{12}$-i monotherapy can be an option also for long-term secondary prevention, where aspirin has been the treatment of choice for many decades and is currently recommended as a first-line treatment [8, 75]. However, the routine use of aspirin in this setting can be questioned based on several considerations: (i) trials establishing the role of aspirin in secondary prevention were performed decades ago, when risk reduction and treatment strategies were not as effective as current ones (e.g., wide use of statins, availability of more potent lipid-lowering agents, advances in PCI and stent technology) [76]; (ii) efficacy and safety of aspirin are not consistent across different settings and could be questioned considering neutral or very small benefits and increased risk of bleeding as compared to no-aspirin or placebo in primary prevention trials [77]; (iii) pharmacodynamic studies have questioned the added benefit of aspirin on top of potent ${\mathrm{P2Y}}_{12}$-i, which may also impact other pathways of platelet activation [26]; (iv) ${\mathrm{P2Y}}_{12}$-i, in particular more potent agents, constitute reliable alternative options that have already affirmed aspirin-free strategies in other contexts (e.g., in PCI patients requiring long-term oral anticoagulation [OAC]) [78].

The first randomized comparison between aspirin and a ${\mathrm{P2Y}}_{12}$-i was performed in more than 19,000 patients with atherosclerotic cardiovascular disease (i.e., recent MI, stroke or symptomatic peripheral artery disease) in the CAPRIE trial. Compared to aspirin, clopidogrel reduced the occurrence of the composite of vascular death, MI, or ischemic stroke (5.32% vs. 5.83%; relative risk reduction 8.7%; 95% CI 0.3 to 16.5; *p* = 0.043) and, despite no difference in bleeding, was associated with a reduced incidence of gastrointestinal hemorrhages (1.99% vs. 2.66%; *p *< 0.002) at a median follow-up of 1.9 years [79]. However, the population differed from that of more recent PCI trials and the 325 mg aspirin dose was higher than that adopted in current practice.

In patients with coronary artery disease, clopidogrel monotherapy was tested over aspirin in patients with stabilized MI or with CCS, with overall neutral results [80, 81]. Similar findings were obtained with ticagrelor monotherapy compared to aspirin in patients undergoing CABG [82, 83]. However, all these trials were small, conducted in heterogeneous patient cohorts and did not avail from the use of current therapeutical standards of care.

A meta-analysis of 42,108 patients with established atherosclerosis from nine randomized trials showed that, compared to aspirin, ${\mathrm{P2Y}}_{12}$-i reduced the risk for MI (odds ratio [OR] 0.81; 95% CI 0.66 to 0.99), without any difference in terms of mortality (OR 0.98; 95% CI 0.89 to 1.08) and major bleeding (OR 0.90; 95% CI 0.74 to 1.10), with consistent findings regardless of the ${\mathrm{P2Y}}_{12}$-i [84]. However, the number needed to treat (NNT) to prevent one MI with ${\mathrm{P2Y}}_{12}$-i monotherapy was high (244 patients), questioning the clinical relevance of these findings.

Some modern day evidence on the use of ${\mathrm{P2Y}}_{12}$-i monotherapy for long-term secondary prevention in patients undergoing PCI with second-generation drug-eluting stents came from a landmark analysis of the GLOBAL LEADERS trial, reporting results between 12 and 24 months, when the trial consisted of a net comparison of ticagrelor and aspirin (Table 4) [19].

**Table 4.** **Long-term ${\mathrm{P2Y}}_{12}$-inhibitor monotherapy**.

	GLOBAL LEADERS landmark analysis	HOST EXAM
Population	All-comer PCI patients who did not experience any adverse event during the first year and who adhered to the assigned treatment (n = 11,121)	East-Asian patients undergoing PCI (n = 5530)
ACS	46%	70%
${\mathrm{P2Y}}_{12}$ inhibitor	Ticagrelor	Clopidogrel
Randomization timing	At the time of PCI (main trial)	Six-to-18 months after PCI
Investigational strategy	${\mathrm{P2Y}}_{12}$-i monotherapy for 12 months	${\mathrm{P2Y}}_{12}$-i monotherapy for 24 months
Control strategy	Aspirin monotherapy for 12 months	Aspirin monotherapy for 24 months
Follow-up	Between 12 and 24 months from randomization	24 months from randomization (30–42 months from PCI)
Primary outcome(s)	Death, or Q-wave MI (adjusted HR 0.74; 95% CI 0.58 to 0.96; *p* = 0.022)	Death, non-fatal MI, stroke, readmission due to ACS or major bleeding (HR 0.73; 95% CI 0.59 to 0.90; *p* = 0.0035)
Bleeding outcome	BARC type 3–5 bleeding (adjusted HR 1.89; 95% CI 1.03 to 3.45; *p* = 0.005)	BARC 2–5 bleeding (HR 0.70; 95% CI 0.51 to 0.98; *p* = 0.036)

Results are presented by reporting the effect of interventional strategy versus reference treatment. Abbreviations: ACS, acute coronary syndrome; BARC, Bleeding Academic Research Consortium; CI, confidence interval; HR, hazard ratio; MI, myocardial infarction; PCI, percutaneous coronary intervention; ${\mathrm{P2Y}}_{12}$-i, ${\mathrm{P2Y}}_{12}$ inhibitor.

This analysis included more than 11,000 patients who did not experience any adverse event during the first year and who adhered to the assigned treatment. Ticagrelor monotherapy significantly reduced the incidence of MACE with respect to aspirin monotherapy (1.90% vs. 2.60%; adjusted HR 0.74; 95% CI 0.58 to 0.96; *p* = 0.022), but this came at the price of a significant increase in BARC bleeding type 3-5 (0.5% vs. 0.3%; adjusted HR 1.89; 95% CI 1.03 to 3.45; *p* = 0.005) [19].

The only randomized head-to-head comparison between clopidogrel and aspirin in patients undergoing contemporary PCI is represented by the multicenter open-label HOST-EXAM trial, which enrolled 5530 East Asian patients who maintained DAPT without adverse events for six-to-18 months after PCI (Table 4). Patients were randomly allocated to either aspirin monotherapy or clopidogrel monotherapy [20]. At two years, clopidogrel monotherapy was associated with a significantly lower incidence of NACE (5.70% vs. 7.70%; HR 0.73; 95% CI 0.59 to 0.90; *p* = 0.0035), reflecting reductions in both MACE (3.70% vs. 5.50%; HR 0.68; 95% CI 0.52 to 0.87; *p* = 0.003) and BARC bleeding type 2–5 (2.30% vs. 3.30%; HR 0.70; 95% CI 0.51 to 0.98; *p* = 0.036). However, there was no difference between the two strategies in terms of all-cause death; from a numerical standpoint, clopidogrel monotherapy was associated with numerically increased rates of all-cause death (1.90% vs. 1.30%; HR 1.43; 95% CI 0.93 to 2.19; *p* = 0.101), driven by noncardiac death (1.20% vs. 0.80%; HR 1.47; 95% CI 0.85 to 2.52; *p* = 0.167), mainly cancer-related [20]. This finding should be interpreted with caution due to statistical limitations, in the wait for the HOST-EXAM Extended study that will follow-up patients for a median of 10 years.

Recently, a network meta-analysis of 73,126 patients from 19 studies using DAPT as common comparator showed a potential net clinical benefit of ${\mathrm{P2Y}}_{12}$-i monotherapy over aspirin following DAPT discontinuation in PCI patients, with aspirin increasing the risk of MI as compared to ${\mathrm{P2Y}}_{12}$-i monotherapy (risk ratio 1.32; 95% CI 1.08 to 1.62), without any significant difference in death (risk ratio 1.00; 95% CI 0.80 to 1.26) and major bleeding (risk ratio 1.12; 95% CI 0.82 to 1.53) [85]. Finally, an individual patient data meta-analysis of 24,325 patients from seven randomized trials compared ${\mathrm{P2Y}}_{12}$-i monotherapy and aspirin in patients with established coronary artery disease: compared to aspirin, ${\mathrm{P2Y}}_{12}$-i monotherapy reduced the risk of the composite of cardiovascular death, MI or stroke (5.5% vs. 6.3%; HR 0.88; 95% CI 0.79 to 0.97; *p* = 0.014), mainly driven by a reduction in MI (2.3% vs. 3.0%; HR 0.77; 95% CI 0.66 to 0.90; *p *< 0.001). A similar decrease was noted for NACE (6.4% vs. 7.2%; HR 0.89; 95% CI 0.81 to 0.98; *p* = 0.020), without any significant difference in major bleeding (1.2% vs. 1.4%; HR 0.87; 95% CI 0.70 to 1.09; *p* = 0.23) [86].

Collectively, the evidence from these trials and meta-analyses supports ${\mathrm{P2Y}}_{12}$-i monotherapy as a viable option for long-term secondary prevention in patients undergoing PCI.

## 4. ${\mathrm{P2Y}}_{12}$-Inhibitor Monotherapy in Patients Requiring Oral Anticoagulation

Antiplatelet therapy is also recommended for patients undergoing PCI requiring long-term OAC, with a brief period of triple therapy (i.e., aspirin, ${\mathrm{P2Y}}_{12}$-i plus OAC) followed by a course of dual antithrombotic therapy (DAT), usually with ${\mathrm{P2Y}}_{12}$-i and OAC, and ultimately by lifelong OAC alone [87, 88].

### 4.1 Early Antithrombotic Therapy

In the early phase after PCI, both DAPT and OAC are required. Two RCTs in the era of vitamin K antagonists (VKAs) paved the way to the concept of transitioning from an initial triple antithrombotic therapy to a subsequent DAT [89, 90]. WOEST, a pioneer RCT of aspirin-free strategies, demonstrated that dual therapy with clopidogrel and VKA from the time of PCI was superior to long triple therapy with DAPT plus VKA (for at least one month and up to one year) in reducing bleeding without increasing MACE [89]. The ISAR-TRIPLE trial explored the reduction of triple therapy duration from six months to six weeks and, differently from the WOEST trial, stopped the ${\mathrm{P2Y}}_{12}$-i, concluding with negative results [90]. Considerations from these RCTs informed the design of subsequent trials of DAT versus triple therapy as “aspirin-free” investigations.

Four RCTs investigated DAT with clopidogrel and a direct oral anticoagulant (DOAC; i.e., rivaroxaban, dabigatran, apixaban, edoxaban) following a short course of triple therapy (randomization time from PCI from zero to 14 days across trials) [91]. The PIONEER AF-PCI showed a reduction in one-year clinically relevant bleeding with DAT (rivaroxaban 15 mg once daily plus a ${\mathrm{P2Y}}_{12}$-inhibitor) as compared to VKA-based triple therapy without a significant difference in terms of ischemic outcomes [92]. In the RE-DUAL PCI trial, DAT with dabigatran 110 mg twice daily was superior to triple therapy in terms of major or clinically relevant nonmajor bleeding, while DAT with dabigatran 150 mg twice daily resulted to be noninferior to triple therapy in bleeding reduction, regardless of clinical presentation [93, 94]. The AUGUSTUS trial implemented a 2 × 2 factorial design to evaluate the relative contributions of DOAC versus VKA and DAT versus TAT: compared to VKA, apixaban reduced the incidence of major or clinically relevant nonmajor bleeding and the composite of death or hospitalization; in addition, dropping aspirin reduced the rates of bleeding, without any increase in death or hospitalization nor in the composite ischemic endpoint [95]. Finally, the ENTRUST-AF PCI trial showed noninferiority, but no superiority, of edoxaban-based DAT versus VKA-based TAT in terms of bleeding, without any significant difference in terms of ischemic endpoints [96]. Collectively, these trials showed that DOAC-based DAT outperformed long-term VKA-based triple therapy in terms of bleeding reduction without evident drawbacks in ischemic protection [78].

### 4.2 Long-term Antithrombotic Therapy

Two RCTs questioned the role of antiplatelet therapy in patients requiring OAC beyond one year after PCI [97, 98].

In the OAC-ALONE noninferiority trial, prematurely terminated due to slow enrolment, OAC alone failed in proving noninferior to DAT in terms of one-year MACE [97].

The AFIRE trial, comparing rivaroxaban monotherapy to DAT with rivaroxaban and an antiplatelet agent, was stopped early because of increased mortality in the DAT group: at a median follow-up of 24 months, rivaroxaban monotherapy was noninferior to DAT for ischemic events and superior for bleeding [98].

The external validity of these RCTs is limited due to the enrolment of East-Asian patients, the high prevalence of VKA adoption in the OAC-ALONE, and the use of rivaroxaban doses not approved for stroke prevention in the AFIRE trial.

## 5. Guidelines

Based on the evidence stemming from RCTs and meta-analyses, both European and American guidelines yielded recommendations on antithrombotic therapy for the early and long-term secondary prevention after PCI [8, 9, 75, 99, 100].

The 2019 guidelines on CCS by the European Society of Cardiology (ESC) recommended aspirin and clopidogrel for six months after PCI (class of recommendation [COR] I, level of evidence [LOE] A). Due to the lack of solid evidence at the time, there were no recommendations about ${\mathrm{P2Y}}_{12}$-i monotherapy [75]. ESC guidelines on non-ST-segment elevation ACS (NSTE-ACS) recommended DAPT with a ${\mathrm{P2Y}}_{12}$-i on top of aspirin for 12 months (COR I, LOE A), with DAPT shortening by discontinuing the ${\mathrm{P2Y}}_{12}$-i three months after PCI (COR IIa, LOE B) and stopping aspirin after three-to-six months (COR IIa, LOE A) being two viable options for patients at high bleeding risk, based on the results of the SMART-CHOICE and TWILIGHT trials [99].

Similarly to European guidelines, the 2021 guidelines on coronary artery revascularization by the American College of Cardiology (ACC), the American Heart Association (AHA) and the Society for Cardiovascular Angiography & Interventions (SCAI) introduced a recommendation for short DAPT (one to three months) with subsequent transition to ${\mathrm{P2Y}}_{12}$-i monotherapy (i.e., clopidogrel or any ${\mathrm{P2Y}}_{12}$-i for CCS and ACS patients, respectively) to reduce the risk of bleeding (COR 2a, LOE A) [8]. Shortening of DAPT was recommended at an earlier timepoint than in ESC guidelines because of the advent of the STOPDAPT-2 trial.

Regarding long-term secondary prevention, ESC guidelines on CCS recommended lifelong aspirin for patients with a previous MI or revascularization (COR I, LOE A). However, clopidogrel was recommended as an alternative in patients with aspirin allergy or intolerance (COR I, LOE B) or in preference to aspirin in patients with either peripheral artery disease or a history of ischemic stroke or transient ischemic attack (COR IIb, LOE B) [75].

In patients with a concomitant indication for OAC, ESC guidelines on NSTE-ACS and atrial fibrillation recommended a very short triple therapy (i.e., one week) followed by DAT (clopidogrel plus a DOAC) up to six months and then OAC alone (COR I, LOE B) [88, 99]. Similarly, a focused update of the AHA/ACC/Heart Rhythm Society guidelines for the management of atrial fibrillation and an updated North American expert consensus document recommended a periprocedural triple therapy followed by DAT with a ${\mathrm{P2Y}}_{12}$-i (clopidogrel or ticagrelor) and OAC up to 12 months, and finally OAC alone [87, 101]. Of note, all these regimens can be personalized according to the trade-off between the risks of ischemic events and bleeding [87, 88, 99, 101].

## 6. Future Directions

A number of RCTs on ${\mathrm{P2Y}}_{12}$-i monotherapy after PCI are currently ongoing (Table 5 and Fig. 2).

**Table 5.** **Ongoing randomized clinical trials of ${\mathrm{P2Y}}_{12}$-inhibitor monotherapy after PCI**.

Trial	Population	Trial Design	Investigational strategy	Control strategy	Primary outcome
**BULK-STEMI** NCT04570345	Patients with ACS undergoing PCI (n = 1002)	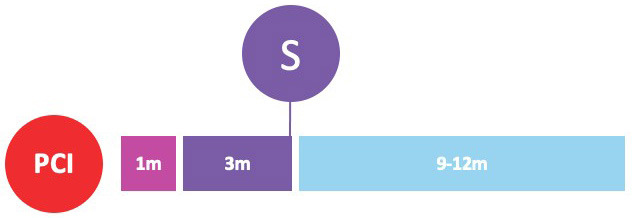	Ticagrelor	DAPT (aspirin plus ticagrelor)	NACE, MACCE and BARC 3 or 5 bleeding at 12 months from randomization
**TARGET-FIRST **NCT04753749	Patients with ACS undergoing PCI (n = 2246)	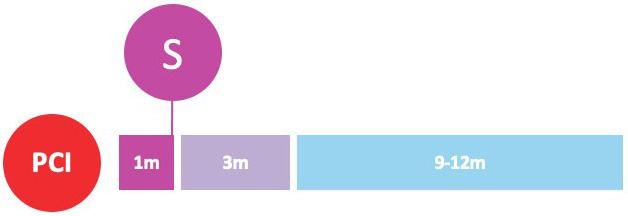	Clopidogrel, prasugrel or ticagrelor	DAPT (aspirin plus any ${\mathrm{P2Y}}_{12}$-inhibitor)	NACCE and BARC 2, 3 or 5 bleeding at 11 months (between one and 12 months from PCI)
**ULTIMATE-DAPT** NCT03971500	Patients with ACS undergoing PCI (n = 3486)	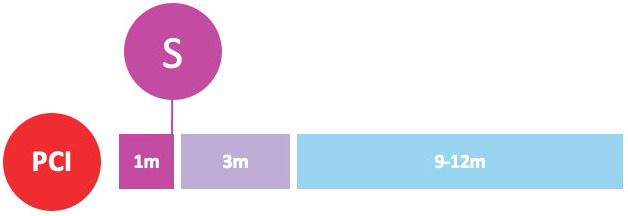	Ticagrelor plus matching placebo	DAPT (aspirin plus ticagrelor)	MACCE and BARC 2, 3 or 5 bleeding at 11 months (between one and 12 months from PCI)
**MATE **NCT04937699	Patients with ACS undergoing PCI (n = 2856)	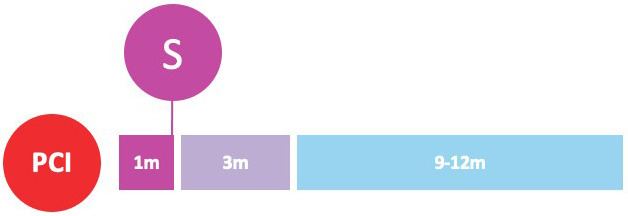	Low-dose ticagrelor followed by clopidogrel	DAPT (aspirin plus ticagrelor)	NACCE at 11 months (between one and 12 months from PCI)
**OPTIMIZE-APT **NCT05418556	Patients with CCS or ACS undergoing intracoronary imaging-guided PCI (n = 3944)	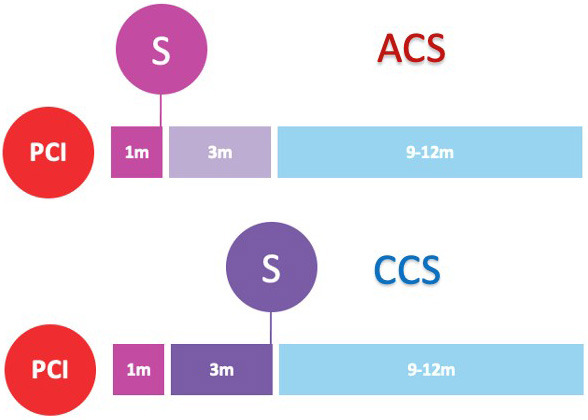	One-month DAPT (aspirin plus clopidogrel) followed by 11-month clopidogrel in CCS; three-month DAPT (aspirin and ticagrelor or prasugrel) followed by nine-month ticagrelor or prasugrel in ACS	One-year DAPT (aspirin plus clopidogrel, prasugrel or ticagrelor, according to clinical setting)	One-year BARC type 2, 3 or 5; one-year NACE; one-year MACE
**NEO-MINDSET** NCT04360720	Patients with ACS undergoing PCI (n = 3400)	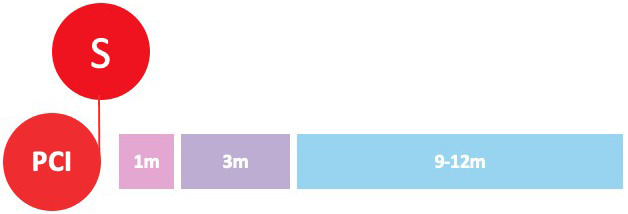	Prasugrel or ticagrelor	DAPT (aspirin plus prasugrel or ticagrelor)	MACCE and BARC type 2, 3 or 5 bleeding at 12 months
**STOPDAPT-3 **NCT04609111	Patients undergoing PCI with ACS or at high risk of bleeding (n = 3110)	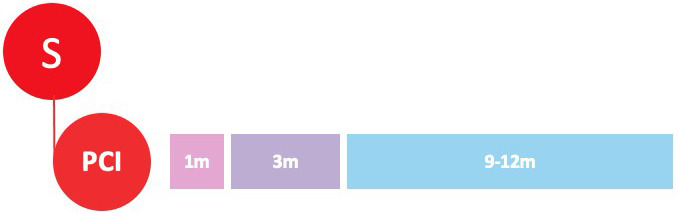	Prasugrel before PCI, followed by clopidogrel one month after PCI	DAPT with aspirin and prasugrel, followed by aspirin monotherapy at one month	MACCE and BARC 3 or 5 bleeding at one month
**OPT-BIRISK** NCT03431142	Patients with ACS undergoing PCI at high risk of both bleeding and thrombosis (n = 7700)	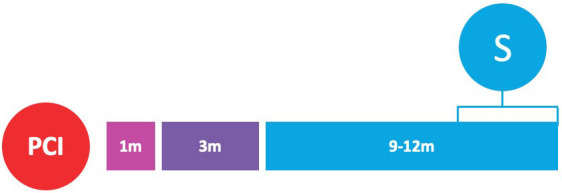	Clopidogrel	DAPT (aspirin plus clopidogrel)	BARC 2, 3 or 5 bleeding at nine months from randomization
**SMART-CHOICE3** NCT04418479	Patients undergoing PCI at high risk of thrombosis (n = 5000)	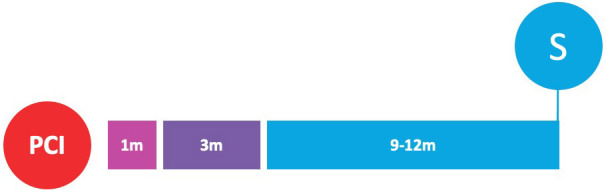	Clopidogrel	Aspirin	MACCE at one year after last patient enrolment
**SMART-CHOICE 2** NCT03119012	Patients undergoing PCI with bioresorbable scaffold implantation (n = 1520)	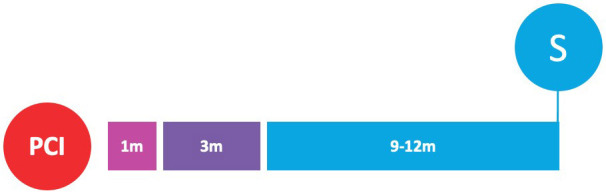	Clopidogrel, prasugrel or low-dose ticagrelor	DAPT (aspirin plus clopidogrel or low-dose ticagrelor)	MACCE at 36 months from randomization

Abbreviations: ACS, acute coronary syndrome; BARC, bleeding academic research consortium; CCS, chronic coronary syndrome; DAPT, dual antiplatelet therapy; m, months; MACCE, major adverse cardiac and cerebrovascular event; MACE, major adverse cardiovascular event; NACCE, net adverse cardiac and cerebrovascular event; NACE, net adverse cardiovascular event; NCT, clinicaltrials.gov number; PCI, percutaneous coronary intervention; S, DAPT shortening timing.

In the setting of ACS, three RCTs are investigating DAPT shortening to one month, followed by a transition to a ${\mathrm{P2Y}}_{12}$-i monotherapy. The TARGET FIRST (NCT04753749) trial is randomizing ACS patients who underwent complete revascularization by PCI one month before to ${\mathrm{P2Y}}_{12}$-i monotherapy (any ${\mathrm{P2Y}}_{12}$-i) or to continue standard DAPT (aspirin plus any ${\mathrm{P2Y}}_{12}$-i) up to 12 months from index PCI; primary endpoints will be net adverse cardiac and cerebral events and BARC 2, 3 or 5 bleeding at 11 months from randomization. Similarly, in the ULTIMATE-DAPT (NCT03971500) trial, patients without adverse ischemic or bleeding events while on DAPT during the first 30 days after PCI for ACS will be randomized to either ticagrelor plus matching placebo or ticagrelor plus aspirin for additional 11 months; the primary endpoints will be MACE and bleeding between one and 12 months [102]. Finally, MATE (NCT04937699) is a multi-step trial enrolling patients without adverse events after one-month DAPT following PCI for ACS; patients will be randomized to a sequential strategy (ticagrelor and aspirin for one month, followed by ticagrelor monotherapy for five months and then clopidogrel monotherapy afterwards) or standard DAPT (ticagrelor and aspirin) for 12 months and will be compared in terms of NACE between one and 12 months.

Transitioning a similar concept to a higher risk setting, the BULK-STEMI (NCT04570345) will randomize patients who completed three months of DAPT after PCI for STEMI to receive either ticagrelor monotherapy or DAPT for additional nine months; the primary endpoints will be NACE, MACCE and major bleeding at one year.

Interestingly, two RCTs are exploring an even more precocious “aspirin-free” approach, consisting of an immediate ${\mathrm{P2Y}}_{12}$-i monotherapy after PCI. The NEO-MINDSET (NCT04360720) trial will randomly allocate ACS patients undergoing PCI to ${\mathrm{P2Y}}_{12}$-i monotherapy (either prasugrel or ticagrelor, stopping aspirin at randomization) or standard DAPT for 12 months, comparing the two groups in terms of one-year MACE and major bleeding. The STOPDAPT-3 (NCT04609111) trial will compare upfront (i.e., starting before the procedure) ${\mathrm{P2Y}}_{12}$-i monotherapy (prasugrel followed by clopidogrel at one month) and one-month DAPT (aspirin and prasugrel) followed by aspirin monotherapy in patients undergoing PCI for ACS or at high-bleeding risk, in terms of both one-month major bleeding and MACE. Notably, the STOPDAPT-3 trial will also provide information on the net comparison of clopidogrel and aspirin monotherapies when DAPT is stopped early (i.e., at one month after PCI).

Several trials are also assessing the role of long-term ${\mathrm{P2Y}}_{12}$-i monotherapy. The OPT-BIRISK trial (NCT03431142) will randomize ACS patients with both bleeding and ischemic risk features who received DAPT for nine to 12 months to clopidogrel monotherapy or DAPT with aspirin and clopidogrel for additional nine months; the primary outcome will be clinically relevant bleeding [103]. As a pure comparison of monotherapies, the SMART-CHOICE 3 (NCT04418479) trial will randomly compare patients who completed 12 months of DAPT to clopidogrel or aspirin monotherapy in terms of MACCE at one year after last patient enrolment. Similar evidence will come from the long-term follow-up (five years) of the STOPDAPT-2 trial that, after the first year, will compare clopidogrel and aspirin monotherapies. Finally, the SMART-CHOICE 2 (NCT03119012) trial will question the optimal therapy in patients implanted with bioresorbable scaffold, randomly assigning those who completed 12 months of DAPT to receive ${\mathrm{P2Y}}_{12}$ monotherapy (clopidogrel or ticagrelor 60 mg twice daily) or DAPT (aspirin plus clopidogrel or ticagrelor 60 mg twice daily) for additional 24 months; the primary outcome will be MACCE at 36 months after PCI.

## 7. Conclusions

In patients undergoing PCI, DAPT with aspirin and a ${\mathrm{P2Y}}_{12}$-i is the treatment of choice to minimize the risk of thrombotic complications. After an initial course of DAPT, the duration of which depends on the clinical setting and the trade-off between ischemia and bleeding, the antiplatelet treatment regimen for secondary prevention consists of a single antiplatelet therapy, traditionally represented by aspirin. Although several investigations are still ongoing, randomized trials accruing over the last few years have shown that shortening DAPT to three or even one month and continuing with ${\mathrm{P2Y}}_{12}$-i monotherapy is an effective bleeding mitigation strategy that does not seem to affect thrombotic or ischemic protection, with the exception of ACS patients, in whom an early transition to less potent ${\mathrm{P2Y}}_{12}$ inhibition with clopidogrel monotherapy was associated with worse net benefit outcomes.

Such data has now been integrated into practice guidelines which now reinforce the evidence on ${\mathrm{P2Y}}_{12}$-i monotherapy recommending their use in patients at high bleeding risk after the shortest possible mandatory period of DAPT. Current knowledge on this topic enables the administration of a ${\mathrm{P2Y}}_{12}$-i monotherapy both in the early phase (i.e., shortening DAPT at one or three months) and for long-term secondary prevention (i.e., after completing the initial DAPT period) in patients undergoing PCI. Ongoing investigations will provide further evidence on timing and specific patient subsets mostly benefiting from this approach.

## Abbreviations

ACC, American college of cardiology; ACS, acute coronary syndrome; ADP, adenosine 5’ diphosphate; AHA, American heart association; BARC, Bleeding Academic Research Consortium; CABG, coronary artery bypass grafting; CCS, chronic coronary syndrome; CI, confidence interval; COR, class of recommendation; COX-1, cyclooxygenase-1; DAPT, dual antiplatelet therapy; DAT, dual antithrombotic therapy; DOAC, direct oral anticoagulant; ESC, European society of cardiology; HR, hazard ratio; LOE, level of evidence; MACCE, major adverse cardiac and cerebrovascular event; MACE, major adverse cardiovascular event; MI, myocardial infarction; NACE, net adverse cardiovascular event; NNT, number needed to treat; NSTE-ACS, non-ST-segment elevation acute coronary syndrome; OAC, oral anticoagulation; OR, odds ratio; PCI, percutaneous coronary intervention; ${\mathrm{P2Y}}_{12}$-i, ${\mathrm{P2Y}}_{12}$ receptor inhibitor; RCT, randomized clinical trial; RR, rate ratio; SCAI, society for cardiovascular angiography & interventions; TRAP-6, thrombin receptor-activating peptide 6; ${\mathrm{TXA}}_{2}$, thromboxane A$A_{2}$; VKA, vitamin K antagonist.
